# Short‐range phenotypic divergence among genetically distinct parapatric populations of an Australian funnel‐web spider

**DOI:** 10.1002/ece3.3084

**Published:** 2017-06-02

**Authors:** Mark K. L. Wong, James D. Woodman, David M. Rowell

**Affiliations:** ^1^ Division of Ecology and Evolution, Research School of Biology Australian National University Canberra ACT Australia; ^2^ National Parks Board Singapore; ^3^ Australian Chief Plant Protection Office Australian Government Department of Agriculture and Water Resources Canberra Australia

**Keywords:** functional traits, mygalomorph, phylogeography, physiology, speciation, Tallaganda

## Abstract

Speciation involves divergence at genetic and phenotypic levels. Where substantial genetic differentiation exists among populations, examining variation in multiple phenotypic characters may elucidate the mechanisms by which divergence and speciation unfold. Previous work on the Australian funnel‐web spider *Atrax sutherlandi* Gray (2010; *Records of the Australian Museum* **62**, 285–392; Mygalomorphae: Hexathelidae: Atracinae) has revealed a marked genetic structure along a 110‐kilometer transect, with six genetically distinct, parapatric populations attributable to past glacial cycles. In the present study, we explore variation in three classes of phenotypic characters (metabolic rate, water loss, and morphological traits) within the context of this phylogeographic structuring. Variation in metabolic and water loss rates shows no detectable association with genetic structure; the little variation observed in these rates may be due to the spiders’ behavioral adaptations (i.e., burrowing), which buffer the effects of climatic gradients across the landscape. However, of 17 morphological traits measured, 10 show significant variation among genetic populations, in a disjunct manner that is clearly not latitudinal. Moreover, patterns of variation observed for morphological traits serving different organismic functions (e.g., prey capture, burrowing, and locomotion) are dissimilar. In contrast, a previous study of an ecologically similar sympatric spider with little genetic structure indicated a strong latitudinal response in 10 traits over the same range. The congruence of morphological variation with deep phylogeographic structure in Tallaganda's *A. sutherlandi* populations, as well as the inconsistent patterns of variation across separate functional traits, suggest that the spiders are likely in early stages of speciation, with parapatric populations independently responding to local selective forces.

## 1 INTRODUCTION

Population divergence is a fundamental basis for the evolution of species and the generation of biodiversity (Rabosky, 2016). Although a differentiation in the genetic material of separate populations is needed for divergence to occur, the process is often influenced, if not dictated by the ecological interactions of individual organisms, which are in turn facilitated by their phenotypic traits (Doebeli & Dieckmann, 2000; Etges, 2014). Thus, where substantial genetic differentiation is known to be present among populations, examining the variation that exists in multiple phenotypic characters may elucidate the factors and mechanisms for their divergence and speciation.

Geographic barriers to dispersal and gene flow promote divergence. In organisms with limited localized dispersal, the potential for divergence is high, and can develop relatively quickly across very fine spatial scales, resulting in short‐range endemism (Harvey, 2002). For instance, spiders in the arachnid infraorder Mygalomorphae (i.e., tarantulas and trapdoor spiders) generally have short‐range dispersal, and are highly susceptible to speciation by vicariant habitat fragmentation and/or parapatric divergence (Bond, Hedin, Ramirez, & Opell, 2001; Leavitt, Starrett, Westphal, & Hedin, 2015). The dispersal of mygalomorphs is limited by their long generation time, ecological niche (e.g., the preference for higher humidity conditions), and inability to use long‐range “ballooning” dispersal strategies (Crews & Hedin, 2006); however, for exceptions see Fisher, Fisher, Skvarla, and Dowling (2014). Dispersal in mygalomorphs is further limited by their desiccation vulnerability, at least partially a consequence of retaining four booklungs that expose large surface areas for water loss (Figueroa, Sabat, Torres‐Contreras, Veloso, & Canals, 2010). Thus, a variety of mygalomorph species exhibit fine‐scale genetic fragmentation and extensive population structuring, making them ideal candidates for studies on population divergence (see Arnedo & Ferrández, 2007; Cooper, Harvey, Saint, & Main, 2011; Hamilton, Formanowicz, & Bond, 2011; Leavit et al., 2015; Satler, Carstens, & Hedin, 2013).

**Figure 1:**
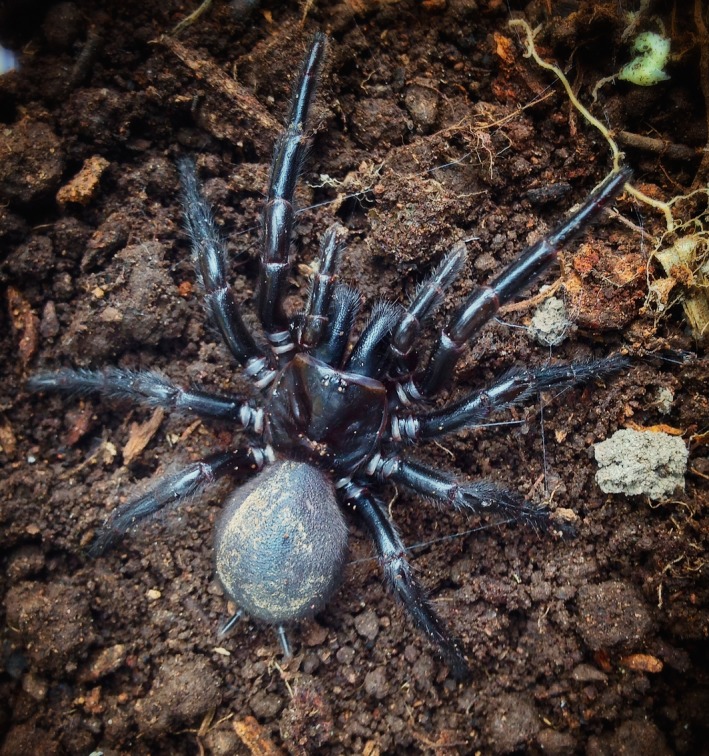
An adult female *Atrax sutherlandi* from Tallaganda forest, New South Wales

**Figure 2:**
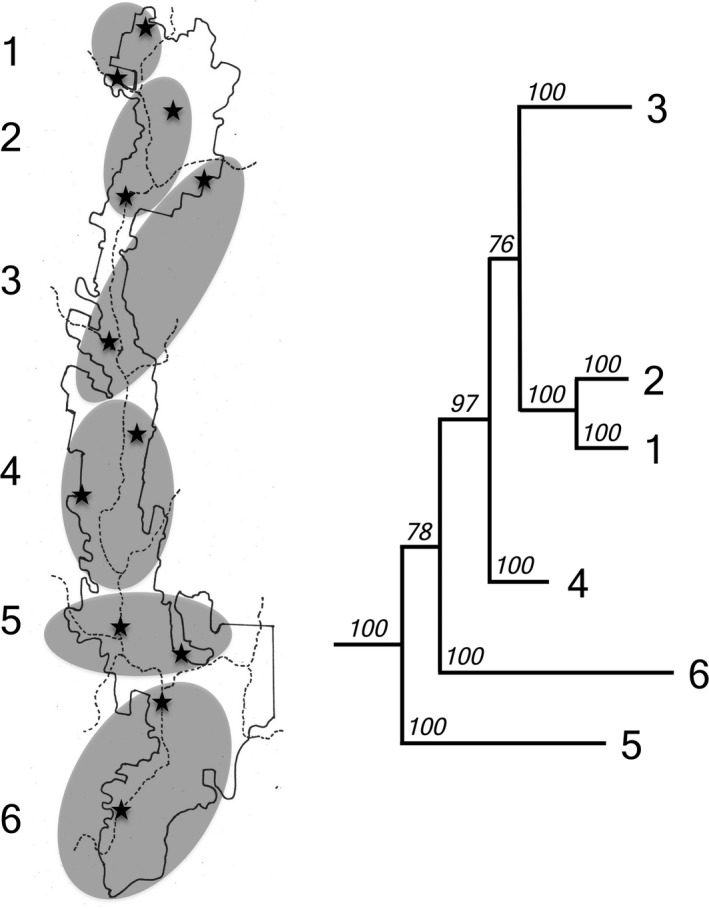
Phylogeography of *A. sutherlandi* at Tallaganda. The *A. sutherlandi* Bayesian phylogeny (right, redrawn from Beavis et al., 2011) separates into six distinct genetic populations, with unequivocal partitioning. These populations are congruent to six parapatric regions in Tallaganda's landscape (left). Stars indicate the locations of 12 collection sites used in the present study, all of which are proximal to sites used in Beavis et al. (2011)

Australian funnel‐web spiders (Mygalomorphae: Hexathelidae: Atracinae) are long‐lived species (>15 years for females based on conservative estimates) and a moist‐adapted forest dwelling group, building web‐lined burrows in soil or rotting logs (Gray, 2010). Our study focuses on the species *Atrax sutherlandi* Gray, 2010 (Figure 1). Phylogeographic analysis on this species at Tallaganda forest, southeastern New South Wales (35°24′S, 36°12′S and 149°28′E, 149°37′E), has revealed extensive patterns of nuclear and mitochondrial divergence, corresponding to six parapatric geographic regions (Beavis, Sunnucks, & Rowell, 2011) (Figure 2). It is proposed that these regions were locations of isolated refugia during the extended climatic shifts of Pleistocene glacial‐interglacial cycling. This is supported by the presence of similar phylogeographic boundaries in five other saproxylic invertebrate species (Beavis et al., 2011; Garrick, Rowell, & Sunnucks, 2012) and one lizard species (Hodges, Rowell, & Keogh, 2007). Furthermore, the extent of genetic divergence among the six regional forms of *A. sutherlandi* (Beavis et al., 2011) is comparable to species‐level differentiation observed in other, morphologically defined, mygalomorph species (Bond et al., 2001; Hamilton et al., 2011; Hedin, Starrett, & Hayashi, 2013).

Here, we investigate phenotypic variation among populations of *A. sutherlandi* at Tallaganda forest. Tallaganda is a continuous, linear strip of forest that extends 110 km in a north/south orientation and 10 km east/west on average. The forest is subject to a smooth rainfall gradient, from 880 mmpa in the north to 1,100 mmpa in the south, and these patterns correlate strongly with soil and log moisture throughout the forest (Woodman, Ash, & Rowell, 2006). Tallaganda's temperatures also follow latitudinal gradients, where the mean ambient temperature gradually decreases from 10.9°C in the north to 9.5°C in the south of the forest (Atlas of Living Australia 2017). According to previous work, the six phylogeographic regions of *A. sutherlandi* (Figure 2) occur as a linear north‐south array (except for minor latitudinal overlap between regions 2 and 3) (Beavis et al., 2011). Although any phenotypic variation observed may be attributed to environmental effects (phenotypic plasticity), or genetic differentiation (local adaption or random drift), the specific phylogeographic patterns of *A. sutherlandi* at Tallaganda may allow for partial distinction between phenotypic variation caused by several likely a priori environmental effects, and that which is produced as a result of genetic effects. Thus, if the phenotypic variation in these moist‐adapted desiccation‐prone mygalomorph spiders is a response to environmental factors, such as humidity and temperature, then its patterns should follow a smooth latitudinal (i.e., clinal) gradient. In contrast, phenotypic variation arising from genetic differentiation will more likely display abrupt and nonlinear patterns of change across the forest.

## 2 MATERIALS AND METHODS

### 2.1 Character selection

We examined variation in metabolic rate, water loss, and a suite of morphological traits for *A. sutherlandi* collected from the six phylogeographic regions at Tallaganda. The character sets were selected a priori based on their functional importance to *A. sutherlandi* life history. Standard metabolic rate and evaporative water loss rate were studied for their potential to respond adaptively to differences in microclimate and behavior. In selecting for morphological traits, two criteria were applied. First, the traits were selected for their accessibility and potential for precise measurement; hence, traits accessible only by extensive dissection (e.g., venom and silk glands) and soft‐structured characters displaying elasticity (e.g., abdomen) were excluded. Second, separate morphological traits were chosen to reflect different aspects of life history, as follows: *trophic organs* may respond to the influence of potential prey item types; *leg morphology* may reflect dispersal patterns; and *spinneret structure* may reflect differences in environmental factors such as soil type and soil moisture acting on burrowing success.

### 2.2 Study sites and specimens

A total of 223 specimens of *A. sutherlandi* were used. These comprised 36 preserved specimens used in the previous genetics study by Beavis et al. (2011) (deposited at the Terrestrial Invertebrate Phylogeography and Population Genetics Laboratory, Research School of Biology, The Australian National University), as well as 187 specimens collected between February and August 2014, from locations within 500 m of the sampling sites of Beavis et al. (2011) (Figure 2). In order to avoid biometric issues associated with sexual dimorphism and developmental allometry, we confined our study to adult females (as per Beavis et al., 2011). All specimens were large and of similar size, while also ensuring that there were no significant differences in body sizes between samples from the six regions. Large adult females were chosen for a number of reasons. First, females are more easily detected and collected as they remain in their burrows, unlike adult males which are vagrant (Bradley, 1993). Second, females are much longer‐lived than males (Levitt, 1961) and so the sex ratio of spiders in the field is heavily skewed to females, making females easier to collect (Wiener, 1959). Finally, juvenile males are indistinguishable from females without dissection, thus choosing females larger than the size range of juvenile males ensured uniformity.

### 2.3 Specimen husbandry

Spiders used for physiological measurements (*n* = 30) were kept at 10°C and 100% RH for 2 months prior to use. For the first 7 weeks, the spiders were fed once per week (between 2000 and 2200 hr) with a medium‐sized (~15 mm) *Tenebrio molitor* larva. One week prior to measurement, feeding was discontinued so as to minimize any variation arising from digestive processes (Anderson, 1970; Mason, Tomlinson, Withers, & Main, 2013).

### 2.4 Measurement of standard metabolic rate

Standard metabolic rate (SMR) was obtained by *V*CO_2_ measurement via flow‐through respirometry at 20°C. We used a differential infrared gas analyzer (LI‐6400XT, Li‐Cor Inc., USA) fitted with a heating circulator (Model: F32‐HL, JULABO Labortechnik GmbH, Germany) for temperature control within a brass animal chamber of an internal volume of 70 cm^3^. All measurements were obtained between 0900 hr and 1600 hr, when the nocturnal spiders are most likely inactive (King, Tedford, & Maggio, 2002). Prior to measurement of SMR, using an electronic balance (sensitivity: 0.1 mg, PB303‐S, Mettler Toledo, Switzerland), the weight (g) of each spider was recorded as a proxy for body mass and body size. The spider was then placed in the dark chamber, and dry ambient air (ca. 0% RH after passing through inline moisture and CO_2_ columns containing Drierite and soda lime, respectively) was passed through the chamber at 100 ml min^−1^. After allowing an hour for acclimation and behavioral settling, SMR (μl CO_2_ h^−1^) was recorded over the next 1‐hr period, in intervals of 5 min. To attain an SMR value that most closely reflected the animal at rest, the average of the three lowest consecutive readings was recorded. Data from spiders that did not settle were discarded; these individuals were re‐measured following a further 5 days in the acclimatization conditions described above. All specimens used for SMR measurement were acclimatized for another week before being used for measurement of evaporative water loss (below).

### 2.5 Gravimetric measurement of evaporative water loss

Total evaporative water loss was measured gravimetrically in a closed system at 20°C and 0% RH. All measurements were obtained between 0900 hr and 1600 hr. Each spider was gently dusted for debris with a fine paintbrush, weighed, and then placed on a perforated stage in a 2‐L glass desiccating jar containing 500 ml of silica gel granules. Temperature was controlled by storing the jar in an incubator (Precision Scientific Model 4, India, Accuracy: 0.1°C). A micro‐datalogger (Hygrochron iButton, DS1923, Maxim Integrated Products, USA) was placed in each jar to monitor temperature and humidity. After 6 hr, the spider was re‐weighed. Data were discarded if the spider excreted during the experimental period. Total evaporative water loss rate (EWLR) (mg H_2_O g^−1^ h^−1^) was calculated on the assumption that mass loss, in the absence of excretion, is equivalent to water loss (Duncan & Lighton, 1994; Edney, 1977). Following SMR and EWLR measurements, the specimens were preserved in 80% ethanol for morphometric measurements.

### 2.6 Morphological traits

**Figure 3:**
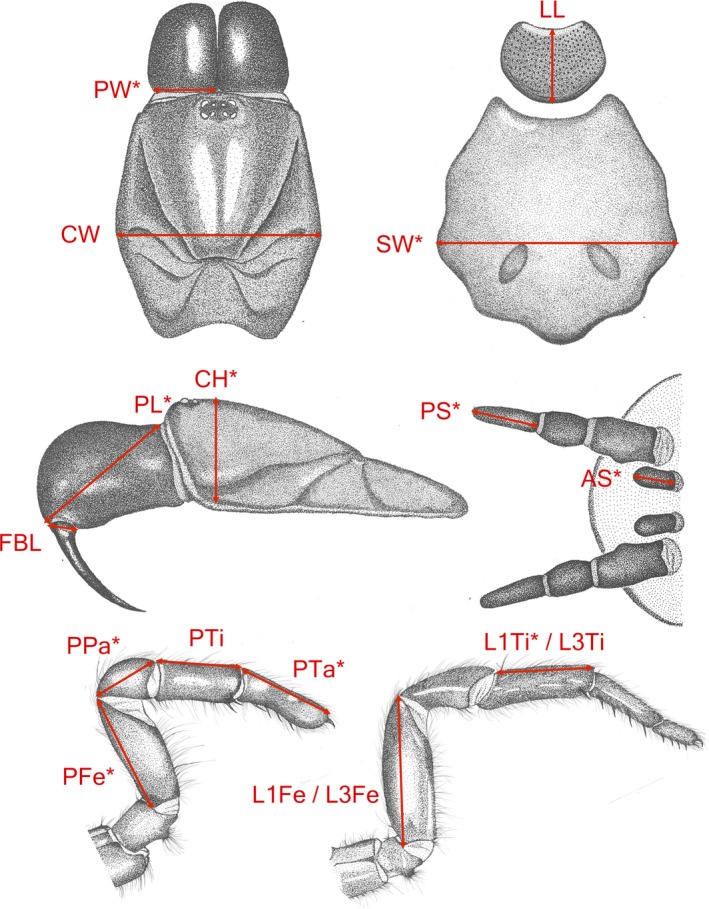
Seventeen morphological traits recorded for all specimens of *A. sutherlandi*. Clockwise from top left, the traits recorded include the Carapace Width (CW) and left Paturon Width (PW) from a dorsal view of the cephalothorax, the Labium Length (LL) and Sternum Width (SW) from a ventral view of the cephalothorax, the lengths of the right Anterior Spinneret (AS) and the last segment of the right Posterior Spinneret (PS) from a ventral view of the abdomen, the lengths of the Femur and Tibia of the first and third legs on the left side (L1Fe, L1Ti, L3Fe, and L3Ti), the lengths of the Femur, Patella, Tibia, and Tarsus of the left pedipalp (PFe, PPa, PTi, and PTa), the Fang Base Length (FBL), Paturon Length (PL), and Carapace Height (CH) from a left side view of the cephalothorax. Ten traits that vary significantly across Regions are indicated (*)

Linear exoskeletal measurements (± 0.001 mm) of morphological traits were obtained using ImageJ (Abràmoff, Magalhães, & Ram, 2004) to analyze images taken by a digital microscope (Model: P‐400Rv, Nikon, Japan). As the major component of variance in morphometric data is often explained through body size (Eberle, Myburgh, & Ahrens, 2014), size correction was applied by dividing all measurements by carapace width (CW) (a measure considered representative of body size in spiders). With size‐corrected measurements of 34 traits from 80 specimens across the six phylogeographic regions, we investigated potentially confounding effects of allometry using the logarithmic transformation technique (Harvey, 1982). Even across the minimized size range used, there was evidence of allometry in some traits (e.g., ocular measurements); these were removed from the analysis. Next, we used a multivariate analysis of variance (MANOVA) to identify 17 traits (Figure 3) which displayed the greatest potential for variation across the regions while incorporating a diversity of functional roles. These 17 traits were subsequently recorded for all 223 specimens and used in statistical analyses.

### 2.7 Statistical analysis

All statistical analyses were performed with SPSS 22.0 (IBM Corp 2013). Linear regressions and analysis of covariance (ANCOVA) were used to investigate the effects of the six phylogeographic regions (henceforth termed “Regions”) on SMR and EWLR. For the 17 morphological traits, multivariate analysis of variance (MANOVA) was used to identify the size‐corrected measurements that varied significantly among Regions. These were then incorporated into a discriminant function analysis (DFA) (Lachenbruch, 1975) to test the extent to which the variation in morphological traits could be used to identify Regions. *Post hoc* Fisher LSD tests were also used to describe the specific relationships between individual Regions for any traits displaying significant variation.

## 3 RESULTS

### 3.1 Standard metabolic rate and evaporative water loss

SMR increases linearly with body mass (*p < *.001, *r* ^2 ^= .671). After controlling for body mass, the ANCOVA shows that SMR does not vary significantly among the Regions (ANCOVA: *p *=* *.127). EWLR also does not vary significantly among the Regions (one‐way ANOVA: *p *=* *.408). The mean SMR and EWLR of *A. sutherlandi* (body mass = 1.19 ± 0.52 g) are 47.12 μl CO_2_ h^−1^ and 9.48 mg H_2_O h^−1^, respectively.

### 3.2 Morphology

**Figure 4:**
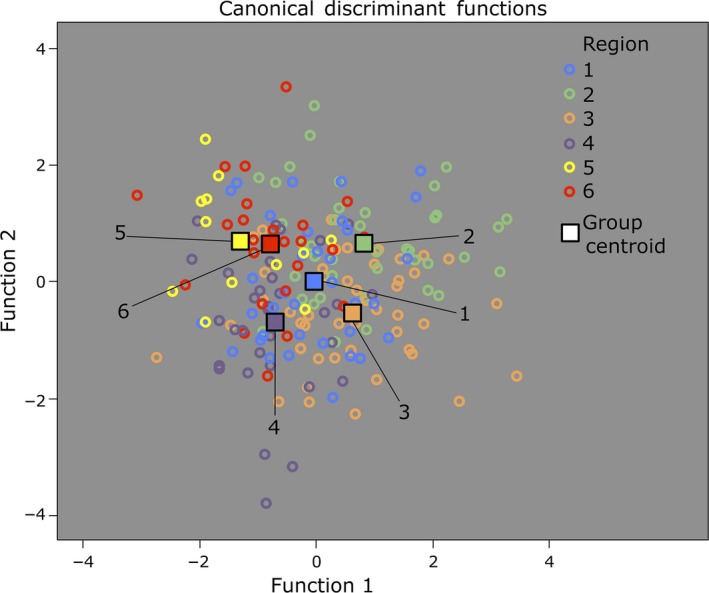
Combined‐groups plot of the first two discriminant functions from the discriminant function analysis (DFA) of the Regions using 11 morphological traits

In all, 10 morphological traits vary significantly for Regions (MANOVA: *p *<* *.05) (Figure 3). A DFA incorporating nine of these traits and two non‐significant traits produces the best reclassification (Figure 4). The first and second functions significantly differentiate the Regions, Wilks’ Lambda = 0.371, χ²(55) = 186.7, *p < *.001, and Wilks’ Lamda = 0.565, χ²(40) = 107.7, *p < *.001, respectively. Overall, the functions achieve a significant 40.9% correct reclassification of cross‐validated grouped cases (C_max_ = 28.4%). Rates of correct reclassification for individual regions are as follows: Region 1 (35%); Region 2 (51.2%); Region 3 (42.2%); Region 4 (48.6%); Region 5 (15.4%); and Region 6 (33.3%).

**Figure 5:**
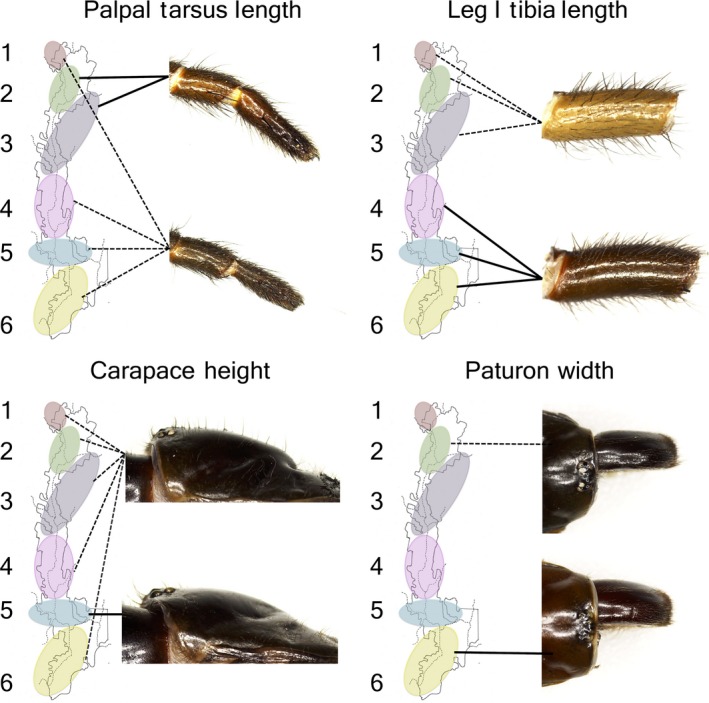
Four morphological traits display distinct and visible structural variation across the Regions. The four traits also have the largest coefficient values on Function 1 of the discriminant function analysis (DFA) (Palpal Tarsus Length: 0.746; Leg I Tibia Length: −0.601; Carapace Height: 0.515; Paturon Width: −0.466). Depending on specific traits, the regions are differentiated into dissimilar grouping combinations. An elongated, muscular palpal tarsus distinguishes Regions 2 and 3 from all other populations. Spiders from Regions 1, 2, and 3 have a stout, straight tibia of Leg I, while an elongated, narrow, and arched form characterizes Regions 4, 5, and 6. The low carapace with a smooth gradient distinguishes Region 5 from all other populations. Paturon width differentiates between Region 2 (short) and Region 6 (wide)

For each of the 10 significant traits from the MANOVA, *post hoc* pairwise comparisons between individual regions were performed using the Fisher LSD. For every trait, the six Regions are classified into three separate groups (“Short,” “Intermediate,” and “Long”), where a Region in any one group has a value which is different from that of Regions in other groups at the 5% level (Table 1). All possible combinations of geographically adjacent Regions (1&2, 2&3, 3&4, etc.) have at least one trait where the two Regions are split into opposite groups (i.e., Short vs. Long), and in four traits (Sternum Width, Anterior Spinneret Length, Palpal Femur Length, and Palpal Patella Length) the maximum and minimum values belong to adjacent Regions (Table 1). Separately, another 4 of the 10 significant traits (Palpal Tarsus Length, Leg I Tibia Length, Carapace Height, and Paturon Width) display visible structural differentiation across the Regions (Figure 5). Statistically, these four traits also best discriminate the Regions as they have the largest coefficient values on Function 1 of the DFA (Figure 5).

**Table 1:** Morphometric trends among the six phylogeographic regions based on a *post hoc* pairwise Fisher LSD for 10 traits with size‐corrected measurements that vary significantly among Regions in the MANOVA

Morphological trait (Size‐corrected)	“Short” regions	“Intermediate” regions	“Long” regions
Carapace height	5	–	1, 2, 3, 4, 6
Sternum width	**5**	2, 3, 6	1, **4**
Paturon width	2	3, 5	1, 4, 6
Paturon length	5	1, 2, 6	3, 4
Anterior spinneret length	**3**, 4	1, 5	**2**, 6
Posterior spinneret length	4, 5	–	1, 2, 3, 6
Palpal femur length	1, **5**	4	2, 3, **6**
Palpal patella length	4, **5**	3	1, 2, **6**
Palpal tarsus length	4, 5, 6	1	2, 3
Leg I tibia length	1, 2, 3	–	4, 5, 6

Based on their mean values for individual traits, the Regions are classified into “Short”, “Intermediate,” or “Long” groups, where a Region in any one group has a value which is different from that of Regions in other groups at the 5% level. For each trait, the Regions with the overall maximum and minimum values have been underlined. All possible combinations of geographically adjacent Regions (1&2, 2&3, 3&4, etc.) have at least one trait where the two Regions are split into opposite groups (i.e., Short vs. Long). For four traits, the maximum and minimum values also occur in adjacent regions (in bold).

## 4 DISCUSSION

In this study, we sought to determine whether variation in three classes of phenotypic characters (metabolic rate, evaporative water loss, and morphological traits) shows patterns of divergence that are consistent with the underlying genetic diversity among phylogeographically distinct populations of *A. sutherlandi*. While examples of fine‐scale phylogeographic structure are not uncommon (e.g., Álvarez‐Presas, Carabyo, Rozas, & Riutort, 2011; Garrick et al., 2004; Milá, Wayne, Fitze, & Smith, 2009; Titus & Daly, 2015; Willett & Ladner, 2009), few studies have examined phenotypic variation among geographically proximate populations, especially among the arachnids. Furthermore, phylogeographic studies investigating phenotype report mixed findings; while some uncover physiological or morphological variation corresponding to genetic differentiation, others report negative results. For example, differentiation in two thermal traits between long‐isolated lizard (Scincidae) populations have been shown to correspond to their phylogeographic divergence (Moritz et al., 2012), and isolated populations of beetles (Tenebrionidae) in the Canary Islands show patterns of morphological diversity which suggest that a widespread common ancestor adapted to the differing habitats of individual islands (Rees, Emerson, Oromi, & Hewitt, 2001). In contrast, little morphological structuring was associated with mtDNA variation in separate haplotype clades of *Bothrops* pit vipers (Puorto et al., 2001), as well as in phylogeographically structured populations of satin bowerbirds (*Ptilonorhynchus violaceus*) (Nicholls & Austin, 2005).

### 4.1 Subterranean burrows may buffer environmental effects on *A. sutherlandi* physiology

Metabolic rates and evaporative water loss rates do not vary significantly among populations of *A. sutherlandi* from their six phylogeographic regions at Tallaganda. The low variation in SMR and EWLR may indicate that climatic variation across Tallaganda's landscape is insufficient to exert strong selective pressures on *A. sutherlandi* populations. The metabolic theory of ecology posits that the majority of variation in metabolic rate is attributed to variation in body size (as indicated by body mass) and environmental temperature (Gillooly, Brown, West, Savage, & Charnov, 2001). Hence, the relatively narrow range of mean annual temperatures across Tallaganda's short 110 km landscape (9.5–10.9°C) (Atlas of Living Australia 2017) may explain the lack of clinal variation in SMR after the large effect of body mass (67.1%) was removed. However, the climatic variation experienced by fossorial spiders like *A. sutherlandi* may be buffered by their sheltered and web‐lined subterranean burrows, as has been reported for the Aganippe mygalomorphs; in arid open claypans humidity levels within the subterranean burrows remain consistently high (Min. Burrow RH = 86.3%) despite drier ambient conditions at the ground surface (Max. Surface RH = 39.5%) (Mason et al., 2013). Similar environmental buffering may explain the lack of variation in EWLR of *A. sutherlandi* in spite of Tallaganda's rainfall gradient (Woodman et al., 2006). Indeed, an absence of female *A. sutherlandi* in pitfall traps (unpublished data) and direct observations from the authors suggest that they rarely, if ever, leave their burrows (cf. vagrant males).

### 4.2 Clinal effects unlikely to account for morphological variation in *A. sutherlandi*

Studies on many taxa including birds, fishes, and insects compare phylogenetic structure with analyses of morphological shape, so as to better understand the strength and nature of selection on species and populations (Barrette, Daigle, & Dodson, 2009; Eberle et al., 2014; Magnan et al., 2014). Often, the results facilitate systematic delineation of taxonomic groups (e.g., Benzoni, Arrigoni, Stefani, & Stolarski, 2012; Bond, Hendrixson, Hamilton, & Hedin, 2012; Mabuchi, Fraser, Song, Azuma, & Nishida, 2014; Pylon & Wallach, 2014). From our analysis, variation is apparent in 10 morphological traits among *A. sutherlandi* populations at Tallaganda. Significant discrimination in the DFA (*p < *.001) indicates that the morphological variation can be explained by the boundaries of six phylogeographic regions although individual regions cannot be accurately distinguished on morphology alone. Notably, all possible combinations of geographically adjacent regions have at least one trait where the two regions are split into opposite groups (i.e., Short vs. Long), and in four traits the maximum and minimum values also occur in these adjacent regions (Table 1). Hence, although the morphological variation among *A. sutherlandi* from different regions is substantial, the absence of clear geographic and clinal trends suggests that the variation is not likely a response (i.e., neither phenotypic plasticity nor local adaption) to factors that vary with latitudinal gradients at Tallaganda (e.g., temperature and humidity). Instead, the disjunct patterns of morphological variation may be a consequence of random genetic drift, or represent the functional responses (adaptive or plastic) of separate populations to currently undetected environmental factors which do not follow latitudinal gradients. Below we make a case for the latter scenario raised, based on our scrutiny of morphological traits that vary the most among the regions, where visible structural differences can plausibly influence the performance of specific functions.

### 4.3 Separate functional responses to multiple independent forces

The 10 traits that vary significantly among the regions (Table 1) do not show consistent patterns of variation (i.e., the regions alternate between the three different groups, depending on the specific trait). Hence, morphological variation in *A. sutherlandi* is unlikely to be driven by a single force acting on the organism as a whole unit. Instead, we propose that multiple independent forces likely act on different morphological traits (e.g., Luquet, Léna, Miaud, & Plénet, 2015). Further illustrating this, four morphological traits show visibly distinct forms among *A. sutherlandi* from Tallaganda, and the grouping combinations of regions produced by each of the four traits are not consistent (Figure 5). Notably, the potential of the four traits for discriminating the regions extends beyond visual observation; it is supported statistically, since these traits also correspond to the four greatest factor loadings on Function 1 of the DFA (Figure 5). It is possible that the morphological variation in *A. sutherlandi* may be explained, at least partially, by the functional responses of different traits to separate independent forces, such as local adaptive pressures. For instance, the structure of trophic organs such as the paturons and carapace may respond to prey type, as the paturons are primarily used for striking and gripping prey (Foelix, 2010; Walker & Rypstra, 2002), and a higher carapace may accommodate larger paturons or facilitate a wider range of motion for the paturons during striking (MKL Wong, pers. obs.). As the legs and pedipalps are used for locomotion and burrowing (Foelix, 2010; Formanowicz & Ducey, 1991; Rovner, 1980), traits associated with these organs may respond to spatial habitat and soil type (but see Bond & Coyle, 1995 for a discussion on the use of the pedipalps in prey capture). As the spinnerets are used for releasing and laying silk, which is in turn used for many different functions in *A. sutherlandi* (e.g., wrapping egg sacs and lining the walls of burrows to guard against desiccation, or laying trip‐lines outside the burrow for prey detection) (Starrett, Garb, Kuelbs, Azubuike, & Hayashi, 2012), traits associated with the anterior and posterior spinnerets may respond to a variety of forces such as climatic factors, soil structure, or prey types.

### 4.4 Dissimilar phenotypic patterns allude to contrasting phylogeography between *A. sutherlandi* and the sympatric species *Hadronyche cerberea*

Another species of funnel‐web spider, *Hadronyche cerberea* L. Koch, 1873, is sympatric with *A. sutherlandi* at Tallaganda. *H. cerberea* is of similar size to *A. sutherlandi*, but burrows in dead wood rather than soil. Crucially, unlike *A. sutherlandi* which shows clear genetic distinctions between phylogeographic regions, *H. cerberea* shows very low genetic diversity and no geographic genetic structuring across Tallaganda (Beavis et al., 2011). It is posited that *H. cerberea* may have become locally extinct during the last glacial maximum before subsequently reinvading Tallaganda. In contrast, *A. sutherlandi* expanded from populations surviving in isolated refugia (Beavis et al., 2011).

In all, 12 of the morphological traits we studied in *A. sutherlandi* were previously examined for *H. cerberea* in Tallaganda (Woodman et al., 2006). In *H. cerberea,* 10 of these 12 traits showed a significant correlation with latitude, with no evidence of disjunct patterns. By contrast, in *A. sutherlandi*, only one of the 12 traits (Leg I Tibia) displays variation that may be interpreted as latitudinal. However, the relationship involves two distinct forms, which distinguish north and south regions, with no intermediates (see Table 1 and Figure 5). Considering their relatively similar biology, the discrepancy between the geographic scale of morphological variation in *H. cerberea* (broader‐scale, clinal) and *A. sutherlandi* (finer‐scale, abrupt) is best accounted by the two species’ disparate levels of underlying genetic diversity; broad‐scale latitudinal gradients of morphological change are associated with low genetic diversity in *H. cerberea*, but fine‐scale patterns of abrupt morphological differentiation are indicative of extensive genetic structure in *A. sutherlandi*.

## 5 CONCLUSION

The clinal effects from temperature and moisture availability gradients on the physiology of *A. sutherlandi* appear to be minimized by the spiders’ behavioral adaptations; there is little evidence of regional differentiation in their water loss and metabolic rate. In contrast, a suite of morphological traits show substantial variation in a disjunct manner, which is congruent to the phylogeographic boundaries of six distinct genetic forms. It appears that changes in the functional morphology of *A. sutherlandi* in each of the six genetically distinct populations occur independently. This independence is highlighted by cases where opposing extremes in morphological structure occur between adjacent populations, suggesting that adjacent forms are responding to different localized forces, or responding differently to the same forces across Tallaganda. If the functional responses of *A. sutherlandi* populations at Tallaganda are indeed adaptive (i.e., not plastic or a result drift), then the species may be in the early stages of speciation. This hypothesis is based on the phylogenetic structure with little gene flow among the *A. sutherlandi* populations, as well as in their potentially recent phenotypic differentiation (as indicated by the current inability to distinguish populations solely based on morphology). Additionally, this would be further supported by the absence of comparable short‐range, disjunct patterns of morphological differentiation in the sympatric and ecologically similar, but genetically more uniform species, *H. cerberea*.

This work has revealed clear relationships between morphological variation and genetic/geographic variation that warrant further investigation through behavioral and life history studies. For example, the functional importance of the morphological variation observed has been inferred from spider behavior, but not directly tested. In the case of the trophic structures examined, although these are clearly used in the processes of prey capture and feeding, the precise impact of a change in size or shape of, say, the paturon, is unclear. This also applies to the function of leg morphology in dispersal and spinneret morphology in burrow construction. In these cases, the nature and impact of these changes may be better addressed through a comparative approach, by comparing these variables across a range of species with different behaviors.

Verification of our findings that populations of *A. sutherlandi* are evolving independently could be achieved through the analysis of males. Adult male morphology, which is markedly different from female morphology, is only achieved at the final molt, and at that point males come under very different selective pressures. Adult males no longer catch prey, eat or burrow and, as vagrant animals, dispersal and defense are likely to be the major factors determining morphology. Indeed, some male characteristics reflect this, most notably longer legs, smaller paturons and more aggressive behavior. Unfortunately, adult males are rarely encountered in the field, and our team has yet to collect sufficient specimens for a thorough statistical analysis (despite sampling at Tallaganda for over 20 years). Finally, future work should also examine the phenology, mating periods, and sexual behavior of *A. sutherlandi* at Tallaganda, which remain unknown for this species and, by extension, the majority of the Atracinae.

## CONFLICT OF INTEREST

None declared.
